# A case report of sodium azide-induced myopericarditis

**DOI:** 10.1093/ehjcr/ytae134

**Published:** 2024-03-20

**Authors:** Constantine Tarabanis, Darcy Banco, Norma M Keller, Sripal Bangalore, Carlos L Alviar

**Affiliations:** Leon H. Charney Division of Cardiology, New York University Langone Health, New York University Grossman School of Medicine, 550 1st Avenue, New York, NY 10016, USA; Leon H. Charney Division of Cardiology, New York University Langone Health, New York University Grossman School of Medicine, 550 1st Avenue, New York, NY 10016, USA; Leon H. Charney Division of Cardiology, New York University Langone Health, New York University Grossman School of Medicine, 550 1st Avenue, New York, NY 10016, USA; Leon H. Charney Division of Cardiology, New York University Langone Health, New York University Grossman School of Medicine, 550 1st Avenue, New York, NY 10016, USA; Leon H. Charney Division of Cardiology, New York University Langone Health, New York University Grossman School of Medicine, 550 1st Avenue, New York, NY 10016, USA

**Keywords:** Sodium azide, Myopericarditis, Colchicine, Case report

## Abstract

**Background:**

Sodium azide exposures are rare but can be lethal as the substance inhibits complex IV in the electron transport chain, blocking adenosine-triphosphate (ATP) synthesis. Sodium azide is mostly used as a propellant in vehicular airbags but is also used in laboratory, pharmacy, and industrial settings. No known antidote exists and its cardiotoxic effects are poorly described in the literature.

**Case summary:**

We describe the case of a 31-year-old patient with major depressive disorder presenting with altered mental status after ingestion of an unknown amount of sodium azide. Although initially chest pain free, she developed pleuritic chest pain 48 h after ingestion. This was accompanied by new diffuse ST elevations on the electrocardiogram and serum troponin elevations concerning for myopericarditis. Treatment was pursued with a 14-day course of colchicine resulting in complete symptom resolution within 4 days of treatment initiation. The patient’s transthoracic echocardiogram was only notable for a preserved left ventricular ejection fraction (LVEF).

**Discussion:**

Cardiac toxicity after sodium azide ingestion usually occurs days after ingestion and has been previously described in the forms of heart failure with reduced ejection fraction complicated by cardiogenic shock. We describe the first case of sodium azide-induced myopericarditis with a preserved LVEF treated with colchicine. Colchicine is an established treatment for pericarditis, but its inhibition of endocytosis, an ATP-dependent cellular function, could be mechanistically relevant to this case.

**Learning points**

- To be able to recognize sodium azide exposure as a potential toxic cause of myopericarditis.
- To understand the effects of sodium azide on cardiomyocyte function.

## Introduction

Sodium azide ingestions are rare with a recent review identifying 156 cases of both accidental and intentional exposures between 2000 and 2020.^1,2^ The substance is mostly used as a propellant in vehicular airbags but has also been used in laboratory, pharmacy, or industrial settings as a reagent probe, biocide, and chemical preservative.^1^ The substance’s widespread availability (even through online retailers) and its prior use in terrorist attacks render this an important toxin for clinicians to be aware of.^1^ No known antidote exists and its cardiotoxic effects are poorly described in the literature.^1,3^

## Case presentation

A 31-year-old female presented to the emergency department (ED) with nausea, emesis, and altered mental status. Past medical history was notable for major depressive disorder treated with escitalopram and bupropion and had no known prior cardiac history. She reported obtaining sodium azide from a laboratory facility and ingesting an unknown amount within 12 h of presentation in a suicide attempt. Admission vital signs were notable for sinus tachycardia to 100 b.p.m., blood pressure of 115/69 mm Hg, temperature of 97.6°F, respiratory rate of 18 breaths per minute, and an oxygen saturation of 98% on room air. On physical exam, the patient had mydriasis and was awake and alert but oriented only to person. A review of systems was negative for fever, chills, cough, shortness of breath, dysuria, syncope, and new rashes. The patient was initially chest pain free; however, she started experiencing intermittent, sub-sternal, dull, non-radiating chest pain exacerbated by deep inspiration during her inpatient admission at 48 h after ingestion.

**Figure 1:**
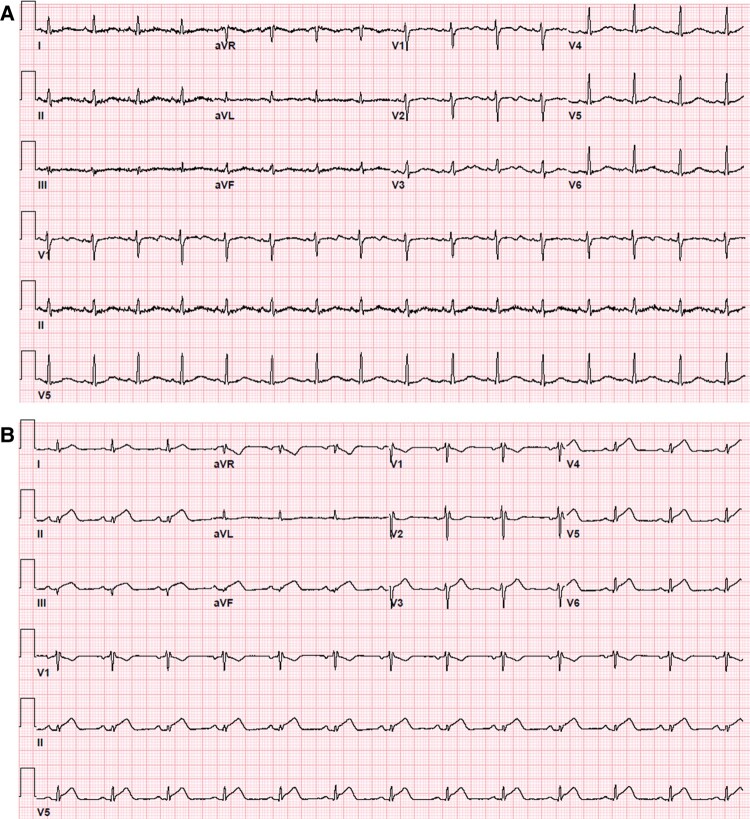
(*A*) The presenting electrocardiogram notable only for sinus tachycardia to 100 b.p.m. and a QTc of 530 ms. (*B*) The electrocardiogram upon development of chest pain with inspiration showing new ST-segment depression in lead aVR and diffuse ST elevations most notable in inferior leads (II, III, and aVF) and V3–6.

**Figure 2:**
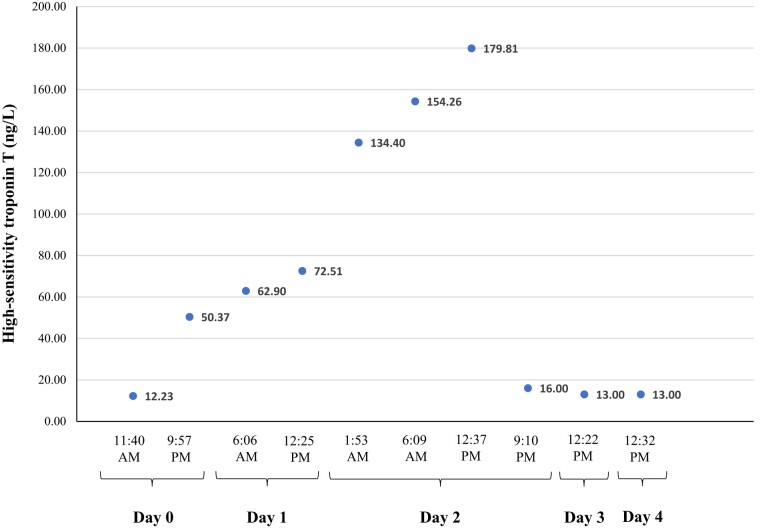
The high-sensitivity troponin-T trend (reference range ≤14 ng/L) from the day of ingestion (Day 0) to normalization (Day 3).

Admission laboratory testing included a venous blood gas with pH of 7.30, CO_2_ of 16 mm Hg, and lactic acid of 15.7 mmol/L. The basic metabolic panel had an anion gap of 25 mmol/L and a serum bicarbonate level of 11 mmol/L, whereas the complete blood count noted a neutrophilic predominant (85.6%) leukocytosis to 14 610/mcL. The hepatic function panel was within normal limits. Nasal swab PCR tests were negative for influenza A/B, Respiratory Syncytial Virus, and SARS-CoV-2. Urine drug testing was negative, and acetaminophen/ethanol/salicylate levels were undetectable. Initial cardiac biomarkers were negative, and the initial electrocardiogram (ECG) was notable for sinus tachycardia to 100 b.p.m. and a QTc of 530 ms. A subsequent ECG performed while experiencing chest pain on hospital Day 2 showed new ST-segment depression in lead aVR and diffuse ST elevations most notable in the inferior leads (II, III, and aVF) and V3-6 (*Figure 1*). High-sensitivity troponin-T peaked at 179.8 ng/L (upper limit of normal ≤14 ng/L) and normalized within 4 days at which point her ECG was back to baseline (*Figure 2*). The C-reactive protein assay peaked at 7.71 mg/L (reference range 0–5.0 mg/L), whereas the erythrocyte sedimentation rate remained negative. A transthoracic echocardiogram was notable for a left ventricular ejection fraction (LVEF) of 50% without focal wall motion abnormalities, normal valvular function, and no evidence of a pericardial effusion.

On presentation to the ED, toxicology was consulted, and the patient was admitted to the medical intensive care unit (ICU) for close monitoring. With the onset of chest pain and the aforementioned ECG changes, the patient was transferred to the cardiac ICU. Treatment was initiated with colchicine 0.6 mg twice daily with as needed ibuprofen for symptomatic relief. The patient noted improvement in the intensity and frequency of chest pain within 24–48 h and complete symptom resolution within 4 days of treatment initiation. Colchicine was administered for a total of 14 days while inpatient and discontinued upon discharge from the hospital given the risk of recurrent suicide attempt with colchicine ingestion. At a 1-month follow-up appointment, the patient remained asymptomatic and no further cardiac testing was pursued.

## Discussion

**Figure 3:**
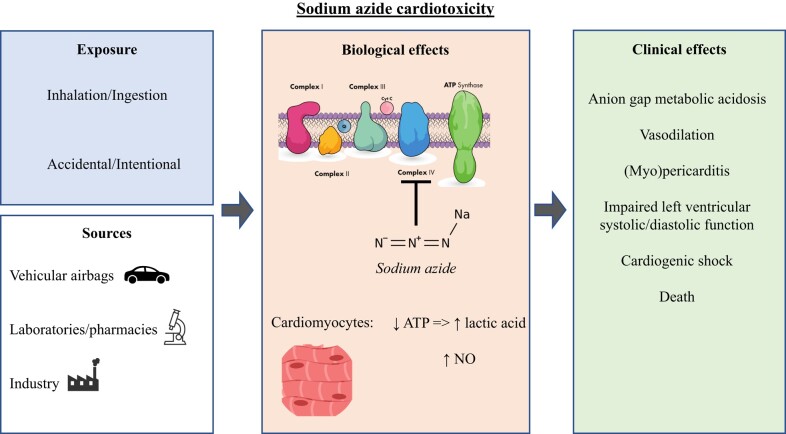
An illustration of the mechanism of sodium azide’s cardiotoxicity.

At low doses, sodium azide ingestion causes dizziness, nausea, and vomiting, whereas at higher doses, it can result in seizures, hypotension, metabolic acidosis, respiratory failure, and death (fatal in doses above 700 mg total or ∼10 mg/kg).^1^ Symptom onset usually occurs within minutes of exposure and no antidote to sodium azide poisoning has been described to date.^1,3^ Sodium azide inhibits the mitochondrial cytochrome C oxidase (complex IV in the electron transport chain) and catalase, blocking adenosine-triphosphate (ATP) synthesis^4,5^ (*Figure 3*). This mechanism is consistent with the patient’s presenting anion gap metabolic acidosis secondary to lactic acid accumulation. Sodium azide can also generate nitric oxide described *in vitro* in erythrocytes, platelets, and isolated blood vessels, resulting in hypotension through a vasodilatory mechanism.^6^

Delayed cardiac toxicity after sodium azide ingestion has been previously described. The aforementioned inhibition of cytochrome C oxidase presumably underlies the substance’s cardiotoxic effects, as cells with high metabolic rates, such as neurons and cardiomyocytes, are particularly susceptible.^1^ One prior case report noted a reduced LVEF to 30% following ingestion that was ascribed to sodium azide-potentiated ischemia given an 80% stenosis detected in the right coronary artery during left heart catheterization.^3^ In that case, no angioplasty was performed and the patient was treated with nitrates with the goal of improving myocardial perfusion with eventual normalization of systolic function within 3 weeks following treatment.^3^ Two case reports noted a reduced LVEF and progression to cardiogenic shock after ingestion of sodium azide requiring vasoactive pharmacotherapy and intra-aortic balloon pump, resulting ultimately in the death of one^7^ and survival of the other patient.^8^ Notably, cardiac histopathologic sections following an autopsy revealed interstitial oedema and myofibrillar degeneration.^7^ In all three cases, myocardial injury was a delayed manifestation as evidenced by troponin elevation at ∼48–72 h after ingestion, similar to the present case report.

Given the pleuritic nature of our patient’s chest pain, suggestive ECG changes, elevated troponin levels, and the absence of other inciting factors, we diagnosed the patient with myopericarditis. The absence of a significantly depressed LVEF in our case, as opposed to prior reports,^3,7,8^ could be secondary to exposure to a lower sodium azide dose in our patient. Given the lack of information on the exact amount ingested, the dose-dependent effect of sodium azide on left ventricular function remains a hypothesis requiring further investigation. Although a prior literature review^9^ listed sodium azide as a potential toxic aetiology of myocarditis, to our knowledge, we describe the first case of sodium azide-induced myopericarditis with a preserved LVEF treated with colchicine. Symptom resolution within 4 days of colchicine initiation could represent either treatment success or the natural progression of the patient’s self-limited course. Colchicine is an established treatment of pericarditis, but its inhibition of endocytosis could be mechanistically relevant to this particular case.^10^ Endocytosis is an ATP-dependent cellular function, so its inhibition by colchicine could have spared ATP, which would be in short supply due to sodium azide’s effect on the electron transport chain. Further *in vitro* and *in vivo* experimentation is required to suggest colchicine as the recommended treatment for sodium azide-induced myopericarditis.

## Conclusions

We describe the first case of sodium azide-induced myopericarditis with a preserved LVEF successfully treated with colchicine. Given the availability of sodium azide and multiple prior exposure reports in the literature, clinicians should be aware of its cardiotoxic effects, while further investigations are required to determine an optimal treatment strategy.

## Data Availability

The data underlying this article are available in the article and in its online supplementary material.

## Lead author biography

Constantine Tarabanis received his Bachelor of Arts degree in Molecular and Cellular Biology from Harvard College and MD degree from Harvard Medical School. He is currently an internal medicine resident at New York University Langone Health. He has previously published basic research on novel fabrication methods for the tissue engineering of collagen-based arterial substitutes. His current research focus is on the application of artificial intelligence algorithms to electrocardiograms.
